# Telephone Triage for Medical Students: An Exploration Through Cultural Historical Activity Theory

**DOI:** 10.1111/tct.70434

**Published:** 2026-04-23

**Authors:** Joe Mawhood, Emily Mackie, Kym Merritt, Judith Donkin, James Fisher

**Affiliations:** ^1^ School of Medicine Newcastle University Newcastle upon Tyne UK; ^2^ Northumbria Healthcare NHS Foundation Trust Northumberland UK

**Keywords:** early clinical experience, medical students, remote consultation, risk, telephone triage, uncertainty

## Abstract

**Background:**

Telephone triage services are widely used in many countries, reflecting a broader shift towards remote patient consultations. Acknowledging this trend, we introduced a programme that placed medical students within a telephone triage service. We present this novel teaching intervention alongside an evaluation of student experiences.

**Approach:**

Attendance at the 111 call centre was mandated for Year 5 medical students and was offered as an optional experience for Year 2 students. Students who had attended a call centre visit were invited to contribute to in‐person focus groups. Three focus groups were conducted, involving 14 students. A semi‐structured interview guide was employed and developed iteratively between focus groups. Data were analysed using thematic analysis. Findings were interpreted through the lens of cultural historical activity theory (CHAT).

**Evaluation:**

Students developed an understanding of how uncertainty and risk are managed in information‐sparse environments. Applying CHAT helped identify tensions within the activity system. These tensions were linked to the tools mediating their learning—specifically, decision‐making algorithms and telephone consultations. Additional tension emerged around the underlying rules of the educational experience and the division of labour within the call‐centre community, which often conflicted with final‐year students' expectations and hindered their learning.

**Implications:**

Students valued the opportunity to gain first‐hand insight into telephone triage and its role within the health system. This setting may offer a useful perspective on managing uncertainty and risk in information‐sparse environments. However, further research is needed to better understand how such placements can be optimised before wider educational adoption is recommended.

## 1 Background

Telephone triage services, such as the UK National Health Service (NHS) 111 service, are established in numerous European countries [1]. Telephone triage is carried out by trained professionals who follow standardised protocols to identify the acuity of a patient's condition, allowing the most appropriate plan for the patient to be determined [2]. Such services exist across a range of healthcare disciplines, most notably midwifery, where telephone triage has provided a critical role in antenatal care for decades [3]. It is recognised that these services can play an important role in managing high volumes of patient contacts [2] but that negative attitudes towards them exist amongst healthcare professionals [4]. The COVID‐19 pandemic drove rapid implementation of remote consultation methods [5], and the use of these methods has continued post‐pandemic. The development of remote consultation skills should therefore be an important part of medical students' undergraduate training. Medical students experience wide‐reaching uncertainty during their training [6], and telephone consultations, due to the lack of visual prompts and cues, can exacerbate their clinical uncertainty [7]. To help students develop skills for managing uncertainty, it has been suggested that educators ought to purposefully introduce specific educational interventions that act as moderators of uncertainty [6]. At our institution, we implemented a programme whereby medical students spent time within a local NHS 111 call centre. In this paper, we present a description of this innovative educational intervention along with the results of a qualitative evaluation, which drew on cultural historical activity theory (CHAT), to understand the experiences of students.

*Telephone consultations, due to the lack of visual prompts and cues, can exacerbate their clinical uncertainty.*

## 2 Approach

### 2.1 Setting

The project was conducted at Newcastle University Medical School, UK. Ethical approval was granted by Newcastle University School of Medicine Research Management Group and Newcastle University Ethics Committee (Ref: 35200/2023).

### 2.2 Design

**FIGURE 1:**
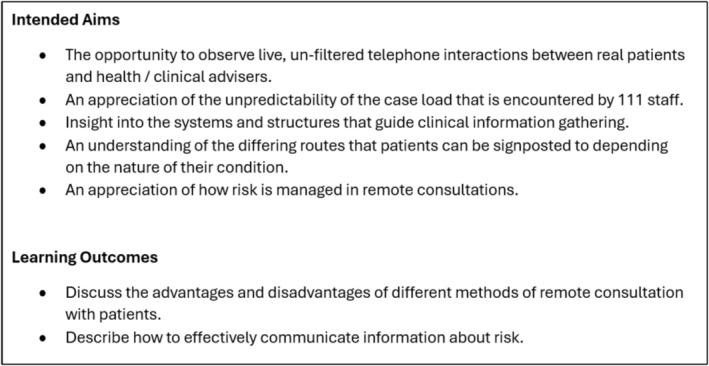
Intended aims and outcomes of the 111 call centre session.

**FIGURE 2:**
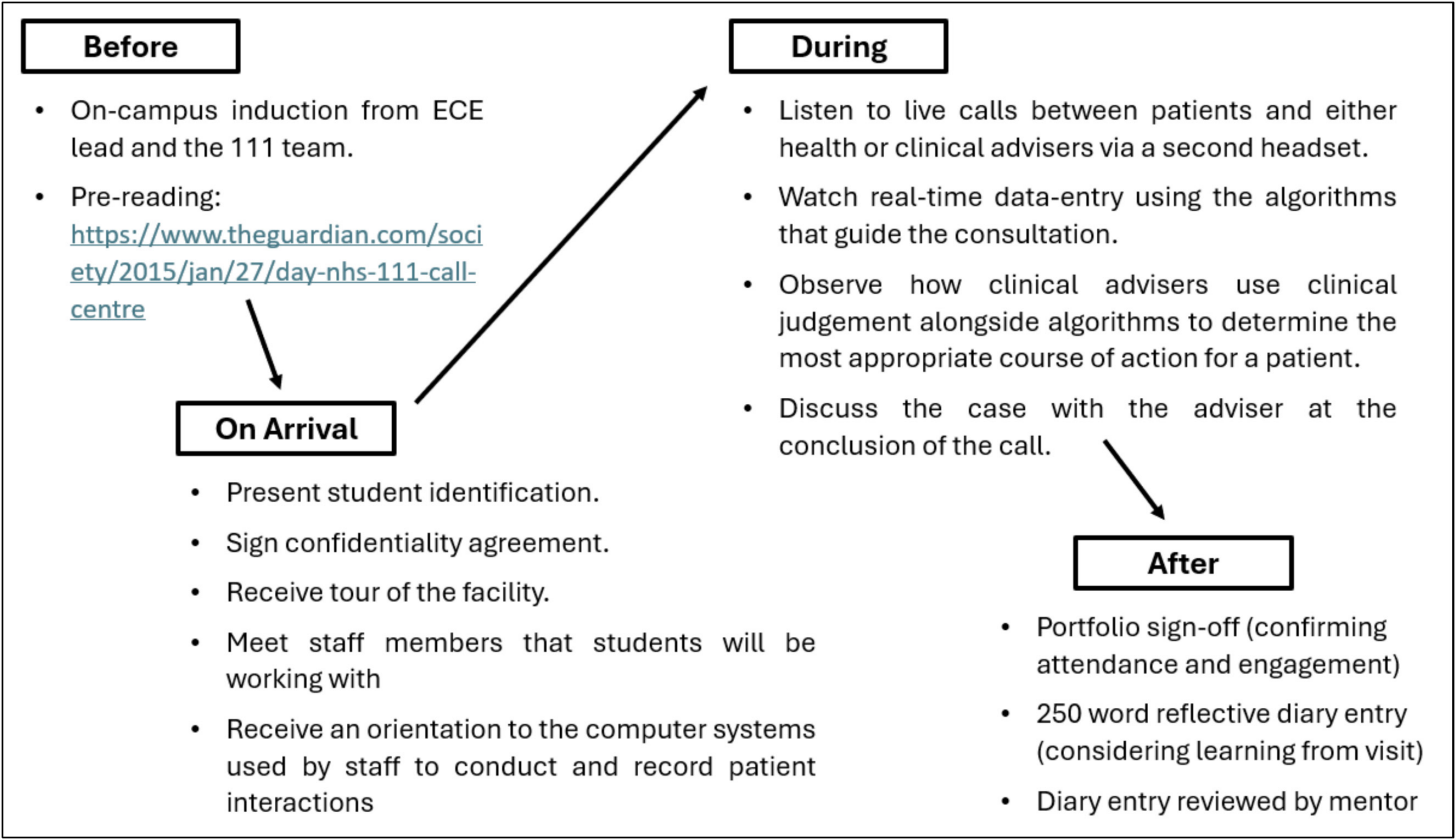
Components of the 111 call centre teaching session (before, on arrival during and after).

Attendance at the 111 call centre was mandated for Year 5 medical students and was offered as an optional, pilot experience for Year 2 students. For Year 2 students, this session formed part of a wider programme of early clinical experience. For Year 5 students, this session formed part of their out‐of‐hours primary care experience. One hundred and forty‐five Year 2 students expressed an interest in attending, with 20 randomly selected to attend. Students spent approximately 4 h at the call centre, during which time they shadowed call‐handlers whilst they interacted with real service users via the telephone. Call handlers were either health advisers (non‐clinical call handlers who follow an algorithm to assess callers) or clinical advisers (trained healthcare professionals who use clinical judgement in conjunction with an algorithm). The aims and outcomes of the session are summarised in Figure 1. The structure of the session (before, during and after) is outlined in Figure 2.

### 2.3 Data Collection

Convenience sampling for focus groups was employed. All Year 5 students and all Year 2 students who attended the pilot visits were invited via email to participate. Ten Year 2 and four Year 5 students were recruited. Two in‐person focus groups of Year 2 students ran (five students per group; nine females, one male) and one Year 5 focus group was conducted (four students; three females, one male). Focus groups were audio recorded, transcribed verbatim and anonymised. Focus group discussion was informed by a semi‐structured interview guide, which developed iteratively between focus groups.

### 2.4 Theoretical Stance

CHAT was chosen as the theoretical lens to interpret our findings. CHAT offers educators a framework for considering learning through practice by unpicking relationships in complex educational settings. CHAT provides a framework to understand how interpersonal relationships and the nature of the environment itself mediate achievement of an object (goal) by learners [8]. The unit of analysis in CHAT, the ‘activity system’, consists of tools, object, subject, rules, community, division of labour and outcomes (see supplementary material). CHAT is particularly helpful where tension and contradictions exist in a system, for example, where learners and healthcare providers are working towards contrasting goals [9].

### 2.5 Data Analysis

Thematic analysis was conducted using Braun and Clarke's framework [10]. J.M. and E.M. familiarised themselves with the dataset, independently coded transcripts and regularly compared codes. Any conflicting interpretations were resolved by J.M. and E.M. with involvement from JF where required. Coding was initially inductive, with participants' experiences shaping this. Thereafter, codes and themes were considered through the conceptual lens of CHAT to refine these, so‐called hybrid inductive–deductive thematic analysis.

## 3 Evaluation

A summary of all themes and codes generated from analysis of focus group data is presented within the supplementary materials. Selected themes of uncertainty and risk, and preconceptions about 111, are presented alongside anonymised illustrative quotes in Tables 1 and 2.

**TABLE 1:** Focus group findings and illustrative quotes: Uncertainty and risk.

Uncertainty and risk	
Students recognised that out of hours triage was challenging, as the call handler lacked detailed information about the patient. This contributed to feelings of uncertainty and altered their diagnostic reasoning process	‘You do not get the big picture … and that might kind of change your thinking’ (Y2, #6)
Students across both year groups were struck by the inability to visualise the patient, and for both groups, this contributed to uncertainty	‘How can you create a clear picture based off just what someone's telling you’ (Y2, #10)
Second‐year students appeared more comfortable being unable to visualise the patient and relied on their imagination. For final‐year students, being unable to visualise the patient led them to express frustration about the nature of telephone consultations, insisting that learning diagnostic reasoning without face‐to‐face contact was not a priority for their learning	‘We're trying to build up an idea in our head of what an unwell patient looks like … I do not really want to learn how to discern if someone's well or unwell based on phone calls’ (Y5, #4)
Students reflected on the calls being brief, isolated encounters with limited opportunity for follow‐up. Feelings of uncertainty were compounded by the unselected nature of the acute problems patients presented with, and the fast‐paced nature of the environment, both of which were new experiences for second‐year students	‘It felt a lot more chaotic and difficult to do, because obviously they were like in the now, rather than telling a story like 3 weeks later’ (Y2, #10)
The decision‐making algorithm employed by call handlers was a source of frustration for students, with it being perceived as restrictive and inflexible	‘I suppose if that's the algorithm then they do not have a choice, like that's the computer, but I think I would struggle to be the call handler in that situation’ (Y2, #9)
Students described dissonance between their own medical decision‐making processes and those of the algorithm, resulting in a sense of discomfort	‘(it) … ends up being so jarring because we are thinking like, you are not doing this the way that we would … your process and your outcome are both possibly very, very, very flawed because you are not using the way that we have been told’ (Y5, #2)
Participants viewed risk as being managed by the algorithm rather than call handlers. They felt that this placed greater responsibility on patients, which conflicted with their developing sense of identity as a doctor	‘Our training as doctors is like we are trained to accept liability for patients’ (Y5, #4)
Students took on passive roles during visits, solely observing consultations. Second‐year students valued the psychological safety and simplicity this role conferred, finding it a helpful space for practising clinical reasoning	‘There's no pressure to actually be right or come up with a diagnosis, it's nice to almost think clinically but not have to say anything’ (Y2, #10)
Final‐year students were frustrated by the prohibition of their involvement, especially when they contrasted with their usual professional roles	‘It was frustrating just to have to sit there and not actually even be allowed to do anything … as a final year we are used to being allowed to go into GP and see patients on our own’ (Y5, #1)
On occasions, final‐year students felt the need to intervene in the phone consultation	‘I sort of politely put my hand across and said like “no no no no, you need to tell them to go to the hospital”’ (Y5, #2)

**TABLE 2:** Focus group findings and illustrative quotes: Preconceptions about 111.

Preconceptions about 111	
Students universally recognised the pressures on 111 and expected negative patient experiences	‘The image I had in my head … like really busy, and like some people on the phones being like angry’ (Y2, #4)
Perceptions of the service were influenced by personal experiences as service users and portrayals in the media, both positive and negative	‘You hear stories about people waiting a long time for calls and then when they get through it's not helpful’ (Y2, #1)
Motivation to attend visits was driven by excitement about the opportunity to witness authentic patient interactions, and a desire to better understand the role the service fulfils	‘I did not fully understand it, [I wanted] to see how it links in with the NHS as a whole and directing to different services and why people end up in A&E after calling 111’ (Y2, #6)
Final‐year students, for whom visits were mandatory, anticipated having a passive role and consequently, were more sceptical about the educational value of the session	‘Low expectations going in … it's just a lot of, like reading from a script, and I thought it was going to be pretty boring to sit and listen to someone read from a script’ (Y5, #3)
Students recounted that these clinical experiences, despite their role as an observer, felt authentic, and generated a strong sense of emotional connection with the patient	‘It made me really upset and I did not even get to like speak to him, but just hearing him’ (Y2, #8)
The experience was perceived as an opportunity to practice clinical reasoning in an authentic setting	‘It was kind of like a clinical reasoning seminar … but like in real life’ (Y2, #10)
Students also recognised that the session provided them with a more nuanced understanding of what the service offers, and how they might interface with it once working as a clinician	‘It's just worth knowing what that service is how it works … it's worth knowing what you are referring someone to’ (Y5, #2)

## 4 Implications

**FIGURE 3:**
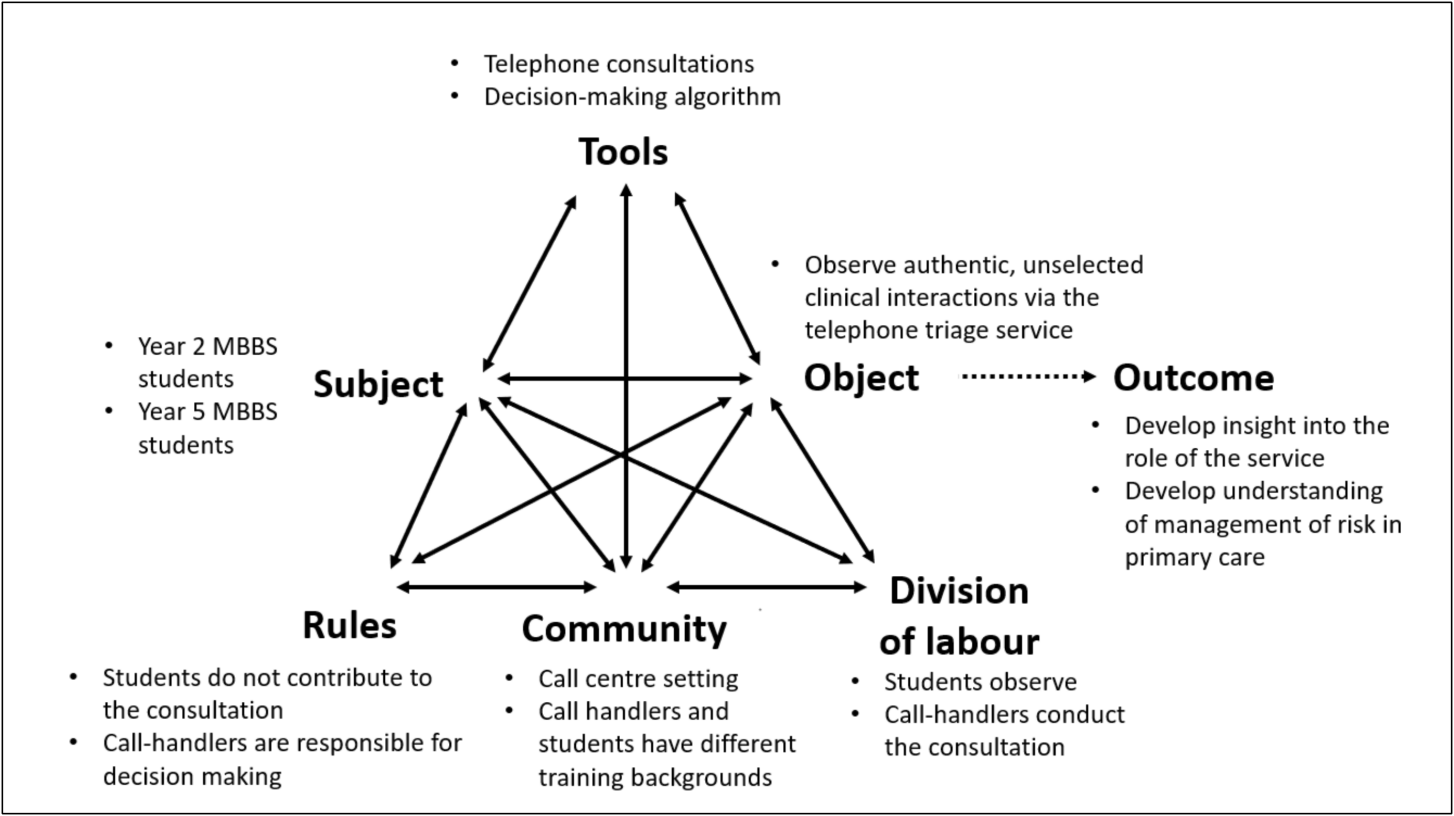
Activity system for medical student learning in the NHS 111 call centre.

Application of CHAT as a conceptual framework enabled understanding of the complex dynamics at play within this learning environment. Factors that undermined learning were identified within the activity system, which is illustrated in Figure 3. The activity undertaken by students within the setting of the call centre was mediated by two tools: first, the telephone, through which the clinical interaction itself was conducted, and second, the decision‐making algorithm, which informed the steps taken by the call handler as the interaction ensued. The use of these tools was mediated by the community in which the activity was conducted, the roles protagonists adopted and the rules that governed these interactions. It is here that tensions arose, or, as CHAT terms them, ‘contradictions’.

*Application of CHAT as a conceptual framework enabled understanding of the complex dynamics at play.*

The telephone introduced an additional layer through which clinical interaction, and therefore learning, was mediated. Clinical uncertainty was experienced due to a confluence of factors—the telephone, the algorithm, the acute nature of interactions, information sparsity, the inability to follow up patients' outcomes and the inability to view the patient. The latter, in particular, proved difficult for students—this finding mirrors previous research that demonstrated students found being unable to draw on clinical examination findings, when conducting remote consultations, disconcerting [7]. Medical students often experience uncertainty during their training [6], and it is recognised that this can generate negative emotions, such as disinterest or aversion [11]. This may explain the response of those students who entirely discounted telephone assessment as a method for assessing an unwell patient. Yet, such a stance is arguably incompatible with present day medical practice. Remote consultations are in widespread use in both primary and secondary care following the COVID‐19 pandemic. Furthermore, in hospital, telephone communication is frequently relied upon when colleagues are communicating with each other about patients' whose conditions have deteriorated. These findings add weight to the argument that greater emphasis ought to be placed on the development of specific telehealth skills during medical school, as well management of risk and uncertainty.

The decision‐making algorithm shaped every patient interaction encountered by students and thus served as a powerful mediator of learning. However, students expressed strong negative views about its use as a consulting tool, which appeared to diminish the quality of their learning experience. Specifically, students were frustrated with the algorithm's seemingly restrictive nature and, at times, disagreed with its recommendations. Some final‐year students perceived that responsibility for decision making, and the associated burden of risk, was abrogated by call handlers onto the algorithm. There was a failure to recognise that the algorithm strives for safe triage, as opposed to accurate diagnostics, and that it is intended for use by call handlers, whose training and skill set differs from medical practitioners. Such dissonance between their own medical decision‐making processes and that of the algorithm were more prominent amongst final‐year students, perhaps because they had more deeply ingrained thinking patterns around how decisions ‘should’ be made. It is notable that students' aversion to the algorithm runs contrary to the fact that most medical decision‐making processes are algorithmic [12] and that clinical guideline use is widespread in modern medicine.

As illustrated in Figure 3, application of the same rules to students with different experience levels created contradictions between the intersection of division of labour and community. Students were required to act as passive observers, deferring to call handlers as facilitators of learning. Second‐year students appreciated the psychological safety provided by not being directly questioned by supervisors, which allowed them to focus more fully on clinical reasoning. This reduced pressure may have lowered intrinsic cognitive load, creating greater capacity for learning. Whilst second‐year students were comfortable adopting a passive, deferential role—and used this position productively—final‐year students found the same passivity frustrating and disengaging. Similar patterns of disengagement have been noted in previous studies of students observing remote consultations [13]. For final‐year students, the proximity to graduation and independent clinical practice created tension, leading to resistance towards the established rules and division of labour within the call centre environment.

The importance of early clinical experience for medical students is well recognised, yet barriers to its widespread implementation are recognised [14]. To increase such exposure, there have been calls to diversify the settings in which early clinical experience is provided [15]. Our work provides an example of a novel setting for valuable clinical experience, and it appears best suited to students at an early stage of their medical training. By attending the session, students developed their understanding of the realities of frontline care provided by the service. They perceived the service to be hardworking, overstretched and struggling to meet patient demand, views that are corroborated in the literature [16]. We acknowledge several limitations to our work. Firstly, our evaluation draws on a small sample of students, and therefore, the generalisability of these findings is limited. Secondly, triangulation of data was not employed. Incorporating data collection via researcher observation of consultations, or by seeking the perspectives of call handlers, would have strengthened our work. The short duration of the visit, coupled with the absence of follow‐up data, means that conclusions cannot be drawn regarding the longer‐term impact of the session. We contend that despite these limitations, our findings do provide readers with context to the innovative session that we present and that the use of CHAT provided a useful lens by which students' experiences could be better understood.

### 4.1 Suggestions for Improvement for This Session

We would recommend a more structured pre‐briefing for students that explicitly addresses the purpose and limitations of the triage algorithm, the distinction between triage and diagnosis and the role of the call handlers. Second, we would recommend that facilitated debriefing follows the session, where groups of students could discuss uncertainty, risk management and the differences between algorithmic and clinical reasoning. These two steps may help students to process the tensions we identified, particularly amongst final‐year students.

Finally, in recognition of the importance of understanding team roles, we would advocate for the integration of an interprofessional education (IPE) element. This might be achieved by positioning call handlers as active educators within the session. This may include structured opportunities for students to engage in dialogue with different members of the 111 team, observe how roles interact in practice and reflect on how responsibilities for triage and risk management are distributed across professional groups. Integrating IPE in this way aligns with the collaborative nature of telephone triage services and may help students develop a more nuanced understanding of team‐based care in remote settings.

## 5 Conclusion

In conclusion, students valued the insights gained from observing the telephone triage service, particularly in understanding its role within the broader healthcare system. These visits contributed to their developing understanding of how uncertainty and risk are navigated in information‐sparse environments, with decision‐making algorithms and telephone consultations serving as key mediators of learning. This setting may offer a useful perspective on managing uncertainty and risk in information‐sparse environments. However, further research is needed to better understand how such placements can be optimised before wider educational adoption is recommended.

*These visits contributed to their developing understanding of how uncertainty and risk are navigated.*

## Author Contributions

**Joe Mawhood:** conceptualisation, investigation, writing – original draft, writing – review and editing, methodology, formal analysis, project administration, data curation. **Emily Mackie:** conceptualisation, investigation, writing – original draft, writing – review and editing, methodology, formal analysis, project administration, data curation. **Kym Merritt:** conceptualisation, writing – review and editing, formal analysis, methodology. **Judith Donkin:** conceptualisation, writing – review and editing, formal analysis, methodology. **James Fisher:** conceptualisation, writing – original draft, writing – review and editing, methodology, formal analysis, supervision.

## Funding

The authors have nothing to report.

## Ethics Statement

Ethical approval was granted by Newcastle University School of Medicine Research Management Group and Newcastle University Ethics Committee (Ref: 35200/2023).

## Conflicts of Interest

The authors declare no conflicts of interest.

## Supporting information

Supplementary Material: The unit of analysis in CHAT – the activity system [arrows represent relationships between constituents of the system].

## Data Availability

Research data are not shared publicly so as to maintain the confidentiality of student participants.
