# Supplementary material for: Telephone Triage for Medical Students: An Exploration Through Cultural Historical Activity Theory
Source: Clin Teach. 2026 Apr 23;23:e70434. doi: 10.1111/tct.70434 (PMC13106924; doi:10.1111/tct.70434)
Supplement: Supplementary file 1 — Supplementary Material: The unit of analysis in CHAT – the activity system [arrows represent relationships between constituents of the system]. [file TCT-23-e70434-s001.docx]

***Supplementary Material: The unit of analysis in CHAT – the activity system [arrows represent relationships between constituents of the system]***

***
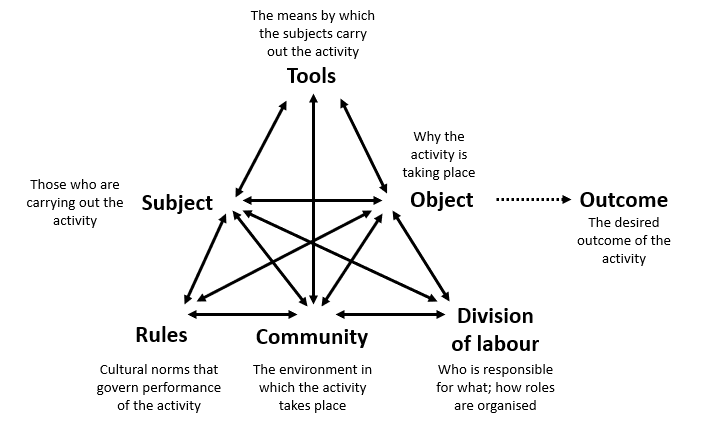
***

***Supplementary Material: Summary of themes and codes
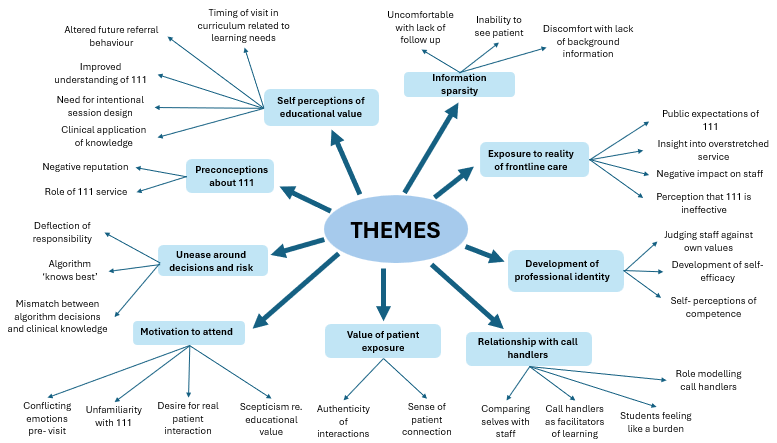
***
